# Prediction model for extrathyroidal extension in thyroid papillary carcinoma based on ultrasound radiomics

**DOI:** 10.1038/s41598-025-19908-5

**Published:** 2025-10-16

**Authors:** Sha-Sha Yuan, Xin-Ran Zhang, Xiao-Qin Yu, Jiao-Jiao Hu, Qing-Qing Chen, Feng Lu, Yang-Jie Xiao, Ying-Fei Huang, Xiao-Hong Fu, Yan Shen

**Affiliations:** 1 School of Gongli Hospital Medical Technology, University of Shanghai for Science and Technology, Shanghai, 200093 China; 2 Ultrasound Department of Gongli Hospital of Shanghai Pudong New Area, New Area, Shanghai, 200135 China; 3 Center of Ultrasonography, Shuguang Hospital, Shanghai University of Traditional Chinese Medicine, Shanghai, 201203 China; 4 Department of Ultrasound, Shengjing Hospital of China Medical University, Shenyang, 110004 Liaoning Province China; 5 School of Optical-Electrical and Computer Engineering, University of Shanghai for Science and Technology, Shanghai, 200093 China

**Keywords:** Papillary thyroid carcinoma, Extrathyroidal extension, Ultrasonography, Radiomics, Oncology, Cancer

## Abstract

This study aimed to construct preoperative prediction models for extrathyroidal extension (ETE) in papillary thyroid carcinoma (PTC) based on ultrasonic radiomics and explore their clinical application value. This retrospective study included PTC patients treated across three centers from 2015 to 2023. Data for 609 cases from two centers were utilized for model construction and divided 4:1 into a training set (*n* = 487; 144 with ETE and 343 without ETE) and test set (*n* = 122; 58 with ETE and 64 without ETE). The external validation set comprised 109 PTC patients from the third center (*n* = 109; 55 with ETE and 54 without ETE). Image features were extracted using Pyradiomics. Feature selection and dimensionality reduction were performed using the least absolute shrinkage and selection operator and principal component analysis to construct radiomics models. Model performance was evaluated by receiver operating characteristic (ROC) curve analysis, and clinical benefit was assessed by decision curve analysis. A total of 806 radiomics features were extracted from the training set data. After feature selection and dimensionality reduction, six significant features were included in the models, including one gray-level size zone matrix feature, one shape feature, one first-order feature, one gray-level run-length matrix feature, and two gray-level co-occurrence matrix features. The extreme gradient boosting (XGB) model showed the best performance in both the test and external validation sets, with area under the ROC curve values of 0.841 and 0.814, respectively. In conclusion, the XGB preoperative ETE prediction model for PTC based on ultrasonic radiomics offers good clinical application value for decision-making regarding therapeutic strategies.

**Supplementary Information:**

The online version contains supplementary material available at 10.1038/s41598-025-19908-5.

## Introduction

In recent years, the incidence of thyroid carcinoma (TC) has increased rapidly worldwide, attracting attention among the medical community^1^. Papillary thyroid carcinoma (PTC) is the most common subtype of TC, accounting for 85%–90% of all TC cases. PTC generally has a favorable prognosis and exhibits low aggressiveness^2^. However, once extrathyroidal extension (ETE), lymph node metastasis (LNM), or distant metastasis (DM) occurs, the prognosis of PTC patients deteriorates significantly^3^. According to the 8th edition of the American Joint Committee on Cancer (AJCC) staging system, extrathyroidal extension (ETE) is defined as the direct extension of papillary thyroid carcinoma into the perithyroidal soft tissues, including the strap muscles, subcutaneous soft tissues, larynx, trachea, esophagus, skeletal muscles, and the recurrent laryngeal nerve^4^. Studies have identified ETE as a key independent predictor of disease-specific survival^5^. Tumor cells in patients with ETE often adhere to or infiltrate surrounding tissues. Compared with cases without ETE, those with ETE require more aggressive treatment strategies, including wider resection margins, and face significantly higher intraoperative risks^6^. Moreover, ETE is associated with significantly reduced cancer-specific survival rates. The 10-year cumulative incidence of remission is 0.0% in PTC patients with extensive ETE, compared with 29.3% in those without extensive ETE^7^. Moreover, PTC with ETE is associated with a higher postoperative local recurrence rate than PTC without ETE^8^. This may be related to residual microinvasion. ETE is also positively correlated with LNM (especially central neck LNM) and distant metastasis to the lungs and bones. Previous studies have confirmed that the risk of distant metastasis is significantly higher in PTC patients with ETE than in PTC patients without ETE and that ETE is an independent risk factor for PTC recurrence^9,10^.

The optimal treatment strategy for TC varies depending on the patient’s specific condition and can include surgery, radioactive iodine therapy, and targeted therapy^11^. Surgery is the primary treatment for TC, especially in early cases with localized lesions. For TC patients with ETE, total thyroidectomy is generally recommended. However, surgery can lead to hypoparathyroidism, causing hypocalcemia, and may also result in damage to the recurrent laryngeal nerve, resulting in hoarseness. The surgical procedure as well as the long-term postoperative recovery can impose psychological stress on patients, leading to anxiety and depression^12^. Therefore, accurate preoperative prediction of ETE is crucial. Unfortunately, the clinical diagnosis of ETE still has certain limitations, as it mainly relies on preoperative imaging examinations and intraoperative pathological assessments. Ultrasonography is the first imaging modality for thyroid evaluation^13^, but its diagnostic accuracy is highly dependent on the skill level of the operator. In recent years, radiomics has made some progress in the field of thyroid cancer, and some studies have used it to predict LNM or benign and malignant classification^14–16^. However, the prediction of ETE is still significantly insufficient, and most of the existing studies are based on small, single-center samples (*n* < 400) or only use a single modeling method, resulting in insufficient generalization ability^17–20^. In addition, previous studies have lacked in-depth research on the mechanism of association between imaging features and tumor invasion behavior. Therefore, the present study aimed to construct and validate a model for preoperative prediction of ETE through multi-center data combined with multiple machine learning algorithms and to explore the potential biological significance of key imaging markers, in order to overcome the shortcomings of past research.

## Materials and methods

### Patients

This retrospective study included 609 patients with PTC from Gongli Hospital and Shengjing Hospital, as well as 109 PTC patients from Shuguang Hospital. All included patients were treated between January 2015 and December 2023. The inclusion criteria were as follows: (1) pathologically confirmed PTC; (2) complete clinical, ultrasonic imaging, and pathological data; (3) clear ultrasonic images that met the diagnostic requirements; (4) no prior treatment before surgery and (5) age ≥ 18 years. The exclusion criteria included: (1) prior treatment before ultrasonic examination, such as radiotherapy or chemotherapy; (2) incomplete or missing data; (3) poor image quality; and (4) other malignancies in addition to PTC. Patients from Gongli Hospital and Shengjing Hospital (Centers 1 and 2; *n* = 609, including 202 with ETE and 407 without ETE) served as the internal dataset and were divided in a 4:1 ratio into a training set (*n* = 487; including 144 with ETE and 343 without ETE) and a test set (*n* = 122; including 58 with ETE and 64 without ETE). Patients from Shuguang Hospital (Center 3; *n* = 109, including 55 with ETE and 54 without ETE) served as the external validation set. This study was approved by the Ethics Committee of Gongli Hospital, Pudong New Area, Shanghai (Ethics Number: GLYY1s2024-043). All methods were performed in accordance with the relevant guidelines and regulations.

## Image acquisition

The ultrasound devices used to collect the images analyzed in this study included the Philips EPIQ 5, Canon Aplio 500, and Siemens AcusonS3000 ultrasound diagnostic systems, with probe frequencies of 5–12 MHz, 5–14 MHz, and 4–9 MHz, respectively. For imaging, patients lay supine on the examination table with a pillow under the neck and the head tilted backward to fully expose the anterior neck region. All examinations were carried out using the following standardized settings: overall gain 60–65 dB, time-gain compensation manually optimized for uniform echogenicity, dynamic range fixed at 60 dB, imaging depth 3–5 cm, and one to two focal zones positioned at the nodule level. Maximum-diameter static images of each target nodule were exported directly in DICOM format.

## Image segmentation and feature extraction

**Fig. 1:**
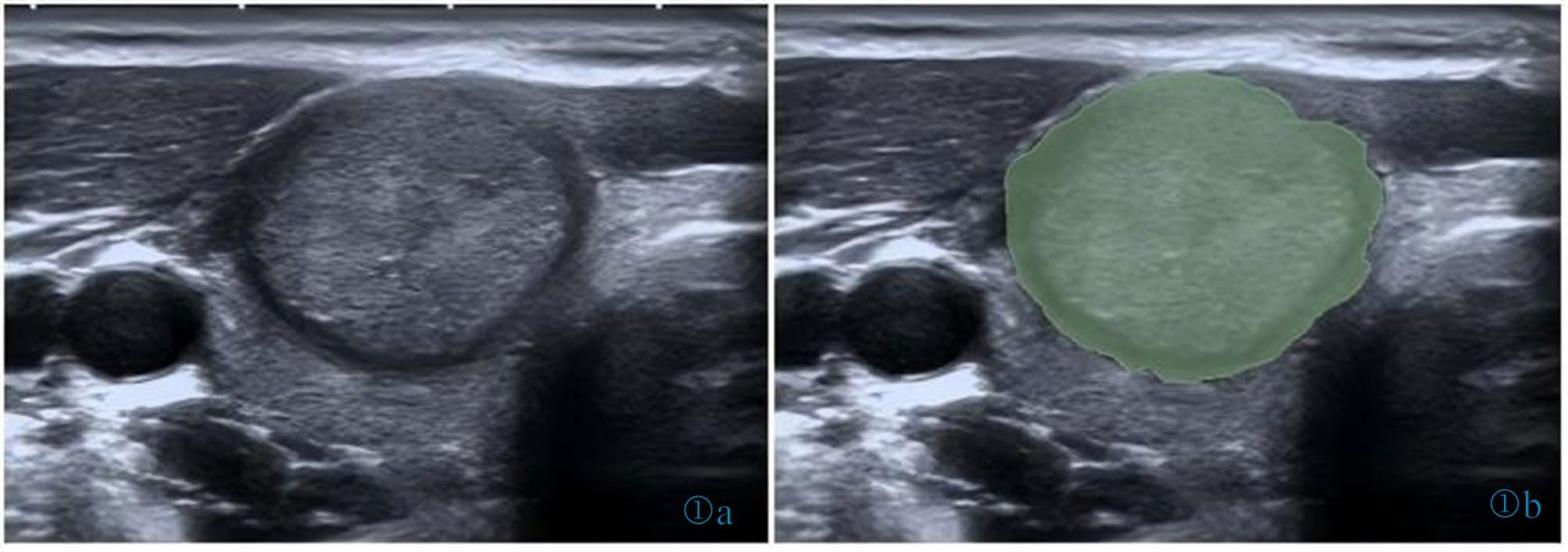
Representative region of interest (ROI) in PTC patient as sketched by 3D slicer software. (a) Original image, and (b) sketched image.

All ultrasonic images in DICOM format were imported into the 3D Slicer software. Two ultrasound physicians manually delineated the margins of the lesions in a double-blind manner (Fig. 1). If discrepancy occurred in the delineation of a lesion by the two physicians, the final region was determined through discussion. Radiomic features were extracted using the Pyradiomics Library in the Python 3.7.16 environment.

## Feature selection and model construction

Using the Pyradiomics package, different categories of radiomic features were extracted from the ultrasonic images of the patients in the training set. Subsequently, a second sonographer with ≥ 5 years of experience independently re-segmented the regions of interest (ROIs) for the same lesions. ICC(3,1) based on a two-way mixed-effects model with single rater and absolute agreement was applied to calculate the ICC for all radiomic features; Features with an intraclass correlation coefficient (ICC) > 0.8 were first selected and then normalized (Supplementary Tables 1 and Supplementary Fig. 1). Subsequently, feature selection and dimensionality reduction were performed using the Least Absolute Shrinkage and Selection Operator (LASSO) regression analysis and principal component analysis (PCA). Optimal λ was selected via 10-fold cross-validation based on the minimum mean squared error (MSE). Given the imbalanced distribution of positive and negative samples, random oversampling (ROS) was applied to the training set after the training–test split but before model training to address class imbalance. Radiomics models were established using several classifiers, including K-nearest neighbors (KNN), logistic regression (LR), decision tree (DT), support vector machine (SVM), extreme gradient boosting (XGB), random forest (RF), linear discriminant analysis (LDA), gradient boosting tree regression (GBTR), multilayer perceptron (MLP), and light gradient boosting machine (LGBM).

### Statistical analysis

All statistical analyses and modeling procedures were performed within dedicated software environments. Clinical baseline comparisons and conventional statistics were executed in SPSS 27.0. Normally distributed continuous variables are presented as mean ± standard deviation (x̄ ± s) and compared among groups using one-way analysis of variance (ANOVA). Non-normally distributed continuous variables are expressed as median (25th–75th percentile) [M (P25, P75)] and were analyzed with the Kruskal–Wallis test. Categorical data were compared using the χ² test or Fisher’s exact test, as appropriate. Radiomic feature extraction was carried out with PyRadiomics (Python 3.7.16). LASSO regression, XGBoost, and LightGBM models were constructed by calling the corresponding scikit-learn1.0.2, xgboost1.6.2, and lightgbm3.3.2 Libraries. SHAP-based model interpretation was implemented with the shap 0.42.1 package.

### Model performance evaluation

For the evaluation of model performance, decision curve analysis (DCA) was first conducted to assess the potential clinical utility of the predictive models. Subsequently, five-fold cross-validation was carried out to evaluate the stability and generalizability of the models, to verify consistent performance across different datasets. Finally, the Shapley Additive Explanations (SHAP) approach was utilized within the training set to identify the features that were most influential on model prediction, to thereby enhance the interpretability of the model and provide a comprehensive understanding of its decision-making process. These three approaches were employed to evaluate and validate model performance from multiple perspectives.

## Results

### Patient characteristics

A total of 718 patients from three centers were enrolled. The internal cohort—comprising patients from Center 1 and Center 2—included 609 individuals. The patients from Center 1 included 141 males and 342 females, with a median age of 47.0 years [IQR 35.0–57.0 years; range 18–82 years]. The patients from Center 2 included 30 males and 96 females, with a median age of 50.0 years [IQR 35.0–57.0 years; range 24–77 years]. The internal cohort was randomly split into training and testing sets at a 4:1 ratio. The training set consisted of 134 males and 353 females, with a median age of 47.0 years [IQR 36.0–57.0 years]; the testing set included 37 males and 85 females, with a median age of 48.0 years [IQR 34.0–57.3 years]. The external validation cohort—derived from Center 3—comprised 109 patients (37 males, 72 females) with a mean age of 45.8 ± 12.0 years (range, 18–71 years). Inter-cohort comparisons revealed no significant differences in age (*P* = 0.707) or sex distribution (*P* = 0.383) (Supplementary Tables 2 and Supplementary Table 3).

### Feature selection

**Fig. 2:**
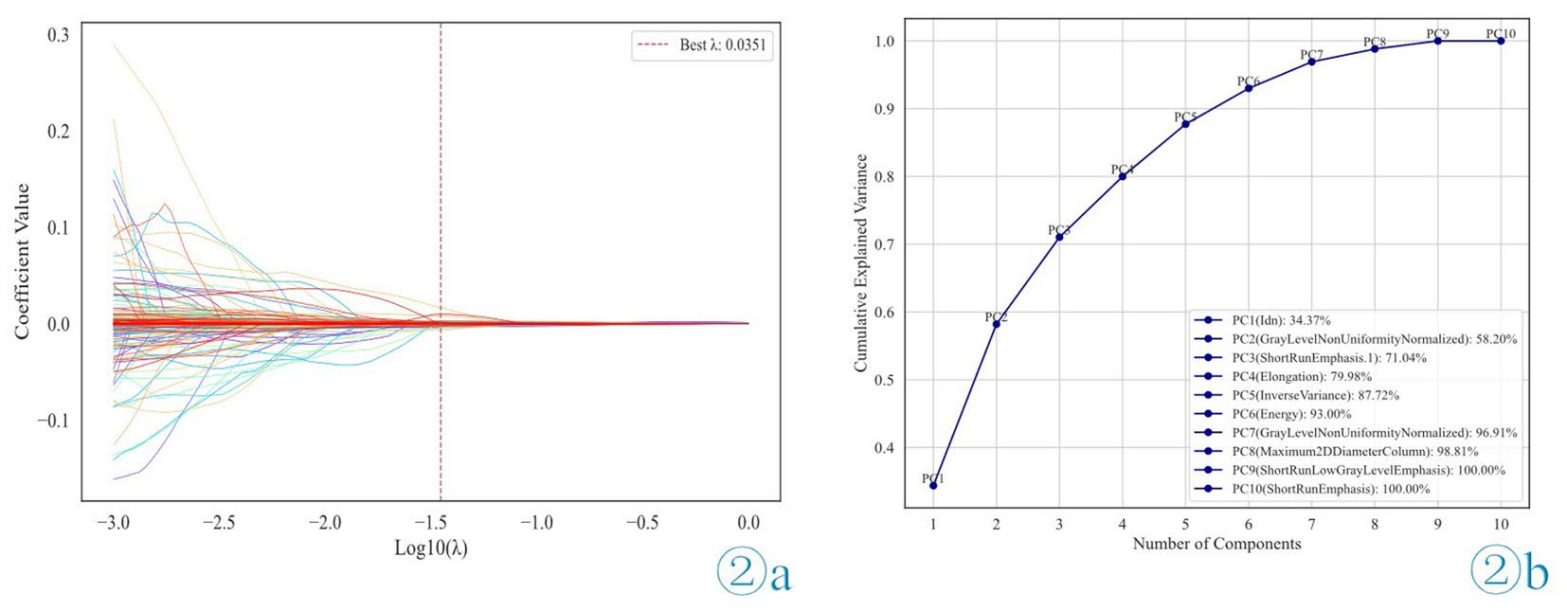
Feature selection. (**a**) LASSO coefficient curves for radiomics features. When λ = 0.0351, the coefficients of most features were reduced to 0. (**b**) Cumulative explanatory variance curve. PC (feature): the variable in parentheses represents the original feature with the highest absolute loading in that principal component analysis.

Using the Pyradiomics package, six categories of radiomic features were extracted from the ultrasonic images of the patients in the training set, including 14 shape features, 162 first-order features, 216 gray-level co-occurrence matrix (GLCM) features, 144 gray-level run length matrix (GLRLM) features, 144 gray-level size zone matrix (GLSZM) features, and 126 gray-level dependent matrix (GLDM) features. The study results showed that when λ = 0.0351, the coefficients for most non-critical features were reduced to zero, and 10 features that contributed to the prediction outcome were selected (Fig. 2a). Analysis of correlations among the selected features revealed a certain degree of redundancy. To eliminate redundancy and improve the computational efficiency of the model, further dimensionality reduction was performed using PCA. The cumulative explained variance threshold was set at 95% to balance the retention of information and model simplification. Ultimately, the following six features were retained: ‘Energy’, ‘ShortRunEmphasis.1’, ‘Idn’, ‘GrayLevelNonUniformityNormalized’, ‘Elongation’, and ‘InverseVariance’ (Fig. 2b) (Supplementary Tables 4 and Supplementary Table 5).

### Model construction and validation

**Fig. 3:**
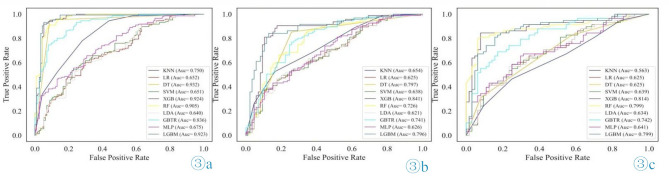
Comparison of ROC curves for each model in the three datasets: (**a**) training set; (**b**) test set; and (**c**) external validation set.

Patients from Centers 1 and 2 were divided into training and test sets at a ratio of 4:1 for the use of training set data to construct different predictive models based on the KNN, LR, DT, SVM, XGB, RF, LDA, GBTR, MLP, and LGBM classifiers. In the training set, the DT model achieved the highest AUC. However, in both the test and external validation sets, the XGB model had the highest AUC. Preliminary results indicated that the DT model performed best in the training set but showed inferior performance and instability in the test and external validation sets. Considering other performance metrics such as accuracy and sensitivity, the XGB model demonstrated the most significant overall net benefit. DCA also revealed that the XGB model had better clinical utility. In addition, the stability and generalizability of the XGB model were evaluated using five-fold cross-validation. The AUC value of this model was 95.1 ± 2.0 in the training set and 87.58 ± 0.75 in the test set. These results further demonstrate the superiority of the XGB model. The ROC curves and performance metrics for each model are shown in Table 1; Fig. 3. The radiomics quality score of this study is 18 points, and additional measures will be implemented in the future to further improve the RQS (Supplementary file 2).

**Table 1:** Comparison of performance metrics for different models in the test set and external validation set.

Group	Model	Accuracy	Sensitivity	Specificity	PPV	NPV	AUC
**Test**	KNN	0.656	0.684	0.624	0.670	0.639	0.654
	LR	0.622	0.579	0.671	0.663	0.588	0.625
	DT	0.800	0.853	0.741	0.786	0.818	0.797
	SVM	0.633	0.547	0.729	0.693	0.591	0.638
	XGB	0.844	0.905	0.777	0.819	0.880	0.841
	RF	0.722	0.663	0.788	0.778	0.677	0.726
	LDA	0.617	0.547	0.694	0.667	0.578	0.621
	GBTR	0.744	0.800	0.682	0.738	0.753	0.741
	MLP	0.622	0.558	0.694	0.671	0.584	0.626
	LGBM	0.800	0.863	0.729	0.781	0.827	0.796
**External validation**	KNN	0.557	0.672	0.453	0.527	0.604	0.563
	LR	0.623	0.672	0.578	0.591	0.661	0.625
	DT	0.615	0.828	0.422	0.565	0.730	0.625
	SVM	0.639	0.638	0.641	0.617	0.661	0.639
	XGB	0.812	0.862	0.766	0.769	0.860	0.814
	RF	0.795	0.879	0.719	0.739	0.868	0.799
	LDA	0.631	0.690	0.578	0.597	0.673	0.634
	GBTR	0.738	0.828	0.656	0.686	0.808	0.742
	MLP	0.639	0.672	0.609	0.609	0.672	0.641
	LGBM	0.795	0.879	0.719	0.739	0.868	0.799

### Feature visualization and interpretation

**Fig. 4:**
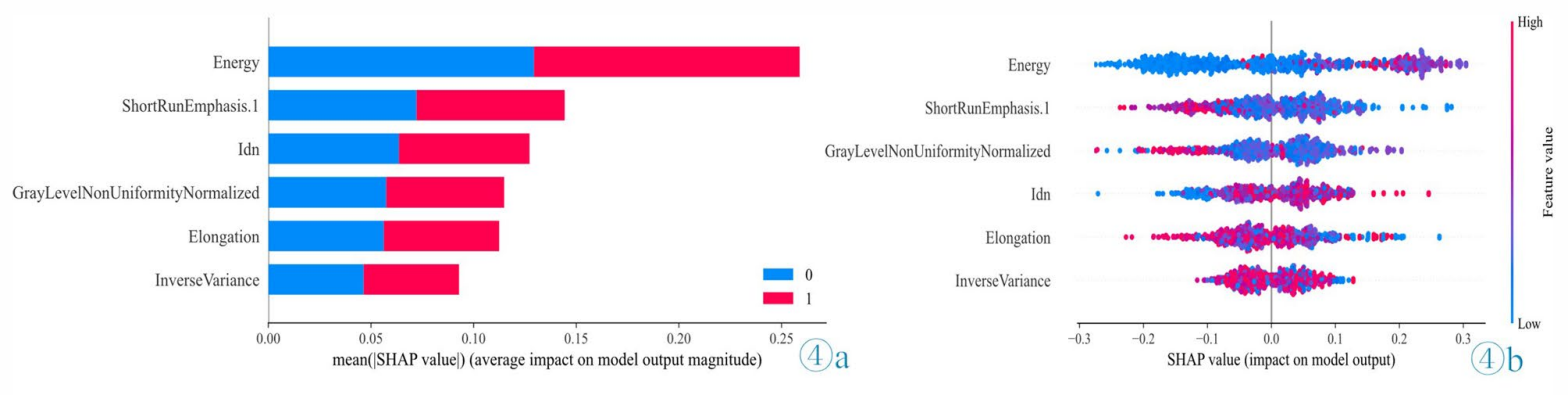
SHAP diagram for the XGB model. (**a**) SHAP bar chart showing the impact of each feature on the XGB model. Category 0: no-ETE; Category 1: ETE. (**b**) SHAP beeswarm plot showing the relationship between the Eigenvalues of the population sample and the predicted results by color.

Based on the aforementioned performance metrics and AUC values, SHAP analysis was employed to provide visual interpretations of the features (Fig. 4). In the feature importance plot, features were ranked according to their mean absolute SHAP value, which reflects their impact on model output. In this study, the feature ‘Energy’ had the highest SHAP value, indicating it had the greatest influence on the prediction outcome. The beeswarm plot provided a more detailed view, illustrating the relationship between the overall feature values in the dataset and their corresponding SHAP values. In these plots, red indicates higher feature values, while blue represents lower feature values. These visualizations facilitate an intuitive understanding of the magnitude of feature values and the relationship between features and prediction outcomes.

## Discussion

ETE is a pivotal prognostic determinant in thyroid carcinoma, specifically PTC. Its deleterious impact is two-fold: (1) it compromises critical anatomical structures, including invasion of the recurrent laryngeal nerve, infiltration of the trachea, esophageal involvement, and encroachment upon the major cervical vessels; and (2) it engenders infiltration of adjacent muscles and soft tissues, thereby escalating the complexity of therapeutic interventions (e.g., surgical resection and postoperative management)^4,21^. Accurate assessment of ETE in PTC patients is crucial for developing individualized treatment plans. Currently, grayscale ultrasound can reveal the presence of ETE to some extent^22,23^. However, research has indicated that the accuracy of ETE diagnosis based solely on grayscale ultrasound features is significantly inferior to that of pathological diagnosis^24^. This limitation may be due to: (1) the high dependence of ultrasound examination on operator experience; (2) the anatomical complexity of the thyroid’s double-layer capsule structure; and (3) the atypical ultrasound imaging features of ETE, which complicate its diagnosis. Radiomics is an emerging, artificial intelligence-based imaging analysis method that can extract a large number of high-throughput features from medical images^25^. Previous studies have shown that ultrasound-based radiomics models represent significant progress in the assessment of TC^15,16,26^. Therefore, the present study aimed to construct a preoperative radiomics model for predicting ETE in PTC patients based on ultrasound imaging and to explore its clinical application value.

From the retrospective radiomics analysis of ultrasound images from 609 patients, six significant features were extracted, including ‘Energy’, ‘ShortRunEmphasis.1’, ‘Idn’, ‘GrayLevelNonUniformityNormalized’, ‘Elongation’, and ‘InverseVariance’. These features serve to distinguish ETE from different perspectives. The features ‘ShortRunEmphasis.1’, ‘GrayLevelNonUniformityNormalized’, and ‘Idn’ reflect the microstructure and complexity within the tumor tissue from a textural perspective, revealing intratumoral heterogeneity and potential invasive behavior. The features ‘Energy’ and ‘InverseVariance’ illustrate the uniformity and heterogeneity of intratumoral gray-level distribution, indicating the growth pattern and internal structure of the tumor. The feature ‘Elongation’ mainly reflects the geometric shape of the tumor, suggesting irregular growth. Combining these different features can improve predictive accuracy for ETE. Based on these six features, we constructed 10 different radiomics models, more than in most other studies. By leveraging the diversity and complementarity of the models, we obtained more stable and accurate prediction results. The results showed that the XGB model offered the best comprehensive predictive performance, with AUC values of 0.924 in the training set, 0.841 in the test set, and 0.814 in the external validation set. A detailed analysis of the model’s architecture revealed that it combines the efficiency of gradient boosting decision trees with regularization techniques. By iteratively adding new tree models to reduce residuals and correct previous errors, the model effectively controls overfitting to achieve enhanced performance. In binary classification tasks, this model has advantages in terms of stability and interpretability.

Consistent with our findings, several early studies also demonstrated the effectiveness of ultrasound radiomics-based models for predicting ETE, reporting AUC values in the range of 0.716–0.832^17–20^. For example, Zhu et al.^17^ also showed the best performance of the XGB model, with an accuracy of 0.77 and an AUC of 0.813, values slightly lower than those in our study, and this difference may be related to the inclusion of a larger sample size (*n* = 337 vs. *n* = 609) in our study. However, the six most important features in their study were all high-dimensional radiomic features, including GLSZM, GLRLM, and GLCM, which highly overlap with the texture analysis features in our study. These texture parameters may indicate that the heterogeneity of tumor margins and local structural destruction caused by microinvasion are the core imaging biomarkers for predicting ETE. Jiang et al.^19^ and Wan et al.^20^ extracted radiomic features from different ultrasound modalities to build models, and their prediction accuracy was higher than that of traditional ultrasound, which further verified the potential of radiomics in ETE evaluation. However, these studies were all single-center studies, which may limit the generalizability of the models and affect the universal applicability of the results. In contrast, our study adopted a multicenter design and introduced external validation to confirm the stability of the model under different device and operator conditions (external AUC = 0.814), reducing the risk of bias for clinical generalization. Notably, Lu et al.^27^ attempted to extracted radiomic features from both two-dimensional and three-dimensional ultrasound images to predict ETE. Among their two-dimensional models, the LR model had the highest AUC of 0.744, but only three models were introduced in the study. In contrast, our study systematically evaluated 10 models to optimize the performance through algorithm diversity. Although the performance of their three-dimensional model was better, the adoption rate of three-dimensional ultrasound is relatively low in some local hospitals, limiting the widespread use of this model.

To eliminate the “black box” phenomenon of radiomics models and increase the transparency and credibility of such models, we further elucidated our model’s decision-making process through SHAP analysis. SHAP values quantify the specific contributions of each feature to a model’s predictions and reflect the sensitivity of the model output to changes in feature values. In our study, we found that ‘Energy’ was the model feature with the highest absolute SHAP value, indicating it has the greatest impact among the model features on the prediction results. This feature primarily integrates wavelet transformation and first-order statistics to extract tumor internal information. By reflecting the overall energy distribution within the tumor, this feature represents the tumor’s internal structure and metabolic activity, thereby aiding in the identification of ETE. This differs from the most influential feature in the model reported by Li et al.^18^, who found that ‘MinorAxisLength’ had the highest absolute SHAP value. However, their study focused on children and adolescents, which may explain the difference from the results of our study in adult PTC patients. ‘Elongation,’ a shape feature, describes the degree of extension of an object in space, and a higher elongation rate may indicate stronger invasiveness. Other texture features also reflect tumor characteristics from different aspects, such as the fineness of image texture, gray-level differences, and gray-level uniformity, providing insight to distinguish ETE in PTC patients. In summary, SHAP analysis can aid in the development an interpretable radiomics models and better identify the associations between radiomics features and PTC, thereby providing more precise guidance for clinical decision-making.

The present study has some limitations. First, we used single-modality ultrasound images and did not comprehensively assess lesions from multiple aspects. Second, the segmentation of lesions by ultrasound physicians is time-consuming. Third, we only used radiomics features to construct predictive models. Previous studies have shown that deep learning also has value in predicting ETE, but we did not compare radiomics with deep learning in this study. Fourth, we were unable to perform further stratified validation by histological subtype (e.g., tall-cell, hobnail variants) or pathological stage.

In conclusion, ultrasound-based radiomics models can play an important role in predicting ETE in PTC patients. By combining external validation and feature visualization, our XGB model offers improved prediction accuracy and a more scientific basis for clinical decision-making.

## Supplementary Information

Below is the link to the electronic supplementary material.

Supplementary Material 1

Supplementary Material 2

## Data Availability

Data supporting the findings of this study are available from the corresponding author upon reasonable request.
