# Supplementary material for: Prediction model for extrathyroidal extension in thyroid papillary carcinoma based on ultrasound radiomics
Source: Sci Rep. 2025 Oct 16;15:36200. doi: 10.1038/s41598-025-19908-5 (PMC12533030; doi:10.1038/s41598-025-19908-5)
Supplement: Supplementary file 1 — Supplementary Material 1 [file 41598_2025_19908_MOESM1_ESM.docx]

**Supplementary Table 1** Features with ICC > 0.8

| Features | ICC |
| --- | --- |
| wavelet-LHH_glrlm_ShortRunEmphasis | 0.817642621 |
| original_shape_Elongation | 0.933991545 |
| original_shape_Maximum2DDiameterColumn | 0.827364334 |
| wavelet-LLH_glrlm_ShortRunEmphasis | 0.874822749 |
| wavelet-HHL_glcm_Idn | 0.901436586 |
| wavelet-HLL_firstorder_Energy | 0.805842639 |
| wavelet-HLL_glszm_GrayLevelNonUniformityNormalized | 0.810730263 |
| wavelet-LLL_glcm_InverseVariance | 0.881776834 |
| wavelet-HHL_glrlm_ShortRunLowGrayLevelEmphasis | 0.825782736 |
| original_glszm_ZoneEntropy | 0.845578278 |
| wavelet-HHL_firstorder_TotalEnergy | 0.853958923 |
| wavelet-HHL_firstorder_Energy | 0.853958923 |
| wavelet-LHL_glszm_SizeZoneNonUniformity | 0.842602778 |
| diagnostics_Image-original_Mean | 0.868911937 |

The intraclass correlation coefficient (ICC) was used to assess feature reliability, ranging from 0 to 1. Thresholds are commonly defined as: ICC < 0.5, poor; 0.5–0.75, moderate; 0.75–0.90, good; and >0.90, excellent. Based on the study design, the ICC(3,1) model (Two-way Mixed, Single Rater, Absolute Agreement) was applied, and features with ICC > 0.8 were retained for further analysis.

**Supplementary Table 2** Comparative analysis of clinical and ultrasound features from three centers

| Variable | Center 1 | Center 2 | Center 3 | χ²/H | p-value |
| --- | --- | --- | --- | --- | --- |
| Age | 47.0(35.0,57.0) | 50.0(35.0,57.0) | 45.8±12.0 | 1.664 | 0.435 |
| Diameter (mm) | 9.20(6.70,14.20) | 8.00(6.00,12.00) | 9.70(6.20,12.95) | 5.899 | 0.052 |
| Sex |  |  |  | 2.953 | 0.228 |
| Male | 141 | 30 | 37 |  |  |
| Female | 342 | 96 | 72 |  |  |
| Composition |  |  |  | 0.644 | 0.725 |
| Mixed cystic-solid | 8 | 2 | 3 |  |  |
| Solid | 475 | 124 | 106 |  |  |
| Echogenicity |  |  |  | - | 0.100 |
| Markedly hypoechoic | 36 | 3 | 3 |  |  |
| Hypoechoic | 438 | 121 | 103 |  |  |
| Iso- or hyperechoic | 9 | 2 | 3 |  |  |
| Shape |  |  |  | 4.367 | 0.113 |
| Wider-than-tall | 191 | 52 | 55 |  |  |
| Taller-than-wide | 292 | 74 | 54 |  |  |
| Margin |  |  |  | - | <0.001 |
| Smooth | 30 | 14 | 20 |  |  |
| ill-defined | 167 | 60 | 10 |  |  |
| Lobulated/irregular | 279 | 52 | 77 |  |  |
| Extrathyroidal extension | 7 | 0 | 2 |  |  |
| Calcification |  |  |  | - | <0.001 |
| Absent or macro-comet tail | 129 | 41 | 33 |  |  |
| Microcalcification | 316 | 84 | 60 |  |  |
| Coarse calcification | 27 | 1 | 16 |  |  |
| Rim calcification | 11 | 0 | 0 |  |  |

“-” indicates Fisher’s exact test

**Supplementary Table 3** Comparative analysis of clinical and ultrasound features from three datasets

| Variable | Training Set | Test Set | External Validation | χ²/H | p-value |
| --- | --- | --- | --- | --- | --- |
| Age | 47.0 (36.0, 57.0) | 48.0 (34.0, 57.3) | 45.8±12.0 | 0.693 | 0.707 |
| Diameter (mm) | 8.8 (6.30, 13.60) | 10.00 (7.45, 14.05) | 9.70 (6.20, 12.95) | 3.355 | 0.187 |
| Sex |  |  |  | 1.921 | 0.383 |
| Male | 134 | 37 | 37 |  |  |
| Female | 353 | 85 | 72 |  |  |
| Composition |  |  |  | - | 0.517 |
| Mixed cystic-solid | 9 | 1 | 3 |  |  |
| Solid | 478 | 121 | 106 |  |  |
| Echogenicity |  |  |  | - | 0.237 |
| Markedly hypoechoic | 28 | 11 | 3 |  |  |
| Hypoechoic | 451 | 108 | 103 |  |  |
| Iso- or hyperechoic | 8 | 3 | 3 |  |  |
| Shape |  |  |  | 7.425 | 0.024 |
| Wider-than-tall | 203 | 40 | 55 |  |  |
| Taller-than-wide | 284 | 82 | 54 |  |  |
| Margin |  |  |  | - | <0.01 |
| Smooth | 42 | 2 | 20 |  |  |
| ill-defined | 181 | 46 | 10 |  |  |
| Lobulated/irregular | 257 | 74 | 77 |  |  |
| Extrathyroidal extension | 7 | 0 | 2 |  |  |
| Calcification |  |  |  | - | 0.005 |
| Absent or macro-comet tail | 141 | 29 | 33 |  |  |
| Microcalcification | 315 | 85 | 60 |  |  |
| Coarse calcification | 23 | 5 | 16 |  |  |
| Rim calcification | 8 | 3 | 0 |  |  |

“-” indicates Fisher’s exact test

**Supplementary Table 4** Principal Component Loading Matrix (PC1–PC10)

| Feature Name | PC1 | PC2 | PC3 | PC4 | PC5 | PC6 | PC7 | PC8 | PC9 | PC10 |
| --- | --- | --- | --- | --- | --- | --- | --- | --- | --- | --- |
| **ShortRunEmphasis.1** | 0.307 | 0.382 | **0.501** | 0.053 | 0.028 | 0.061 | 0.014 | 0.037 | 0.006 | **0.707** |
| **Elongation** | 0.132 | 0.187 | 0.280 | **0.874** | 0.290 | 0.092 | 0.050 | 0.101 | 0.005 | 0.000 |
| Maximum2DDiameterColumn | 0.293 | 0.346 | 0.461 | 0.193 | 0.002 | 0.145 | 0.123 | **0.707** | 0.093 | 0.000 |
| ShortRunEmphasis | 0.307 | 0.382 | **0.501** | 0.053 | 0.028 | 0.061 | 0.014 | 0.037 | 0.006 | **0.707** |
| **ldn** | **0.379** | **0.331** | 0.068 | 0.104 | **0.426** | 0.036 | 0.292 | 0.127 | **0.669** | 0.000 |
| **Energy** | **0.376** | 0.028 | 0.323 | 0.111 | 0.001 | **0.819** | 0.102 | 0.207 | 0.135 | 0.000 |
| **GrayLevelNonUniformityNormalized** | 0.313 | **0.393** | 0.108 | 0.083 | 0.110 | 0.094 | **0.795** | 0.262 | 0.092 | 0.000 |
| **InverseVariance** | 0.231 | 0.298 | 0.008 | 0.312 | **0.766** | 0.025 | 0.402 | 0.055 | 0.095 | 0.000 |
| ShortRunLowGrayLevelEmphasis | 0.366 | **0.384** | 0.089 | 0.040 | 0.296 | 0.186 | 0.270 | 0.091 | **0.711** | 0.000 |
| ZoneEntropy | **0.371** | 0.231 | 0.282 | 0.252 | 0.217 | 0.498 | 0.144 | **0.590** | 0.045 | 0.000 |

Bold values indicate |loading| ≥ 0.30 (threshold for significant contribution). Bold feature names: the six features retained in the final model. Plain feature names: Features that were excluded.

**Supplementary Table 5** Feature Retention Criteria

| Feature | Key Loading Positions | Retention Rationale |
| --- | --- | --- |
| **ShortRunEmphasis.1** | PC3(0.501), PC10(0.707) | Maintained consistently high variance contribution across the multidimensional space |
| **Elongation** | PC4(0.874) | Dominant contribution from a single principal component (core representative of morphological features) |
| **ldn** | PC1(0.379), PC5(0.426), PC9(0.669) | Stable contribution across three principal components, reflecting textural complexity |
| **Energy** | PC6(0.819) | Possesses the strongest explanatory power for the critical variation dimension (PC6). |
| **GrayLevelNonUniformityNormalized** | PC2(0.393), PC7(0.795) | Significant loadings on both the discrete dimension (PC2) and the heterogeneity dimension (PC7). |
| **InverseVariance** | PC6(0.766) | Primary carrier of spatial correlation |


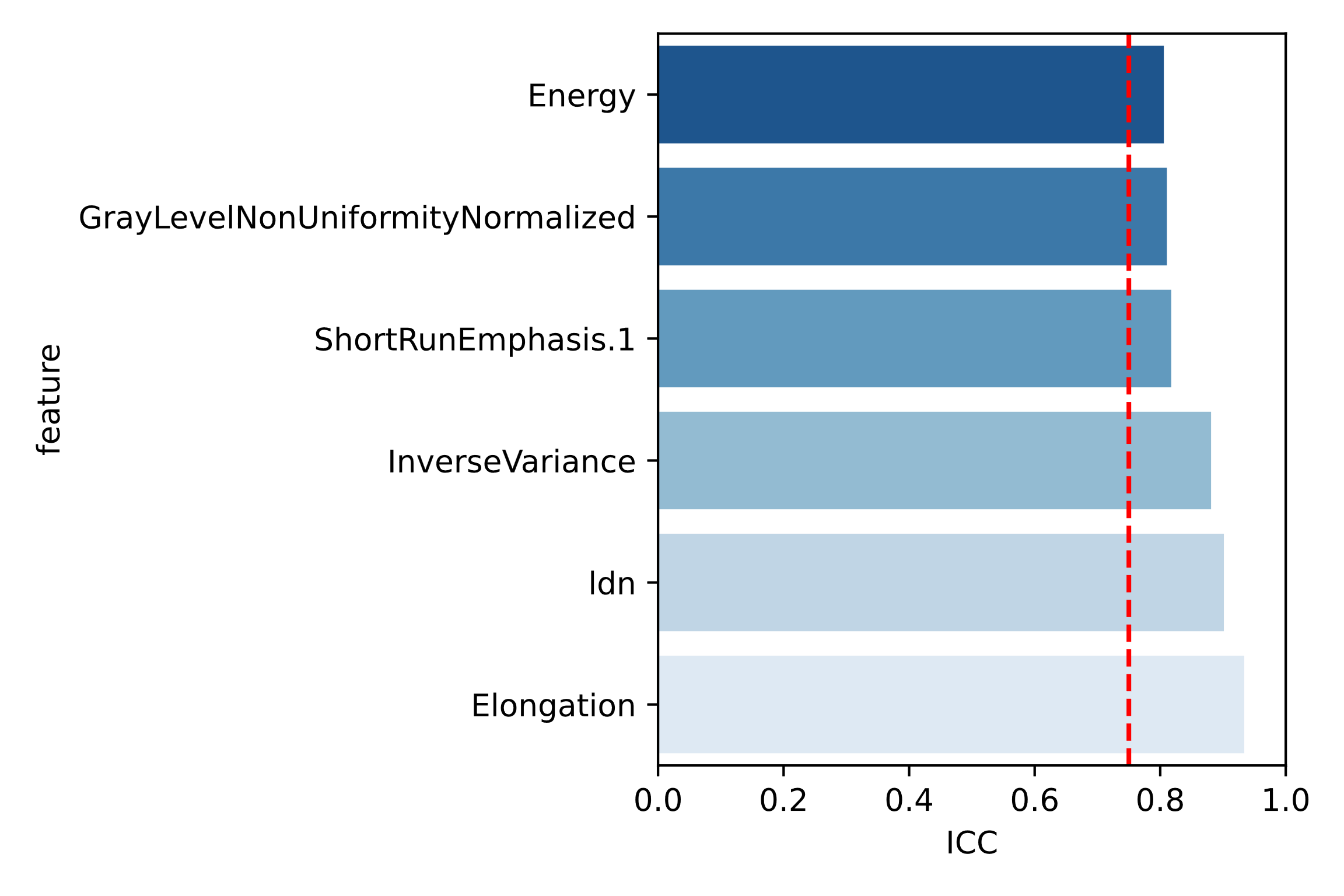

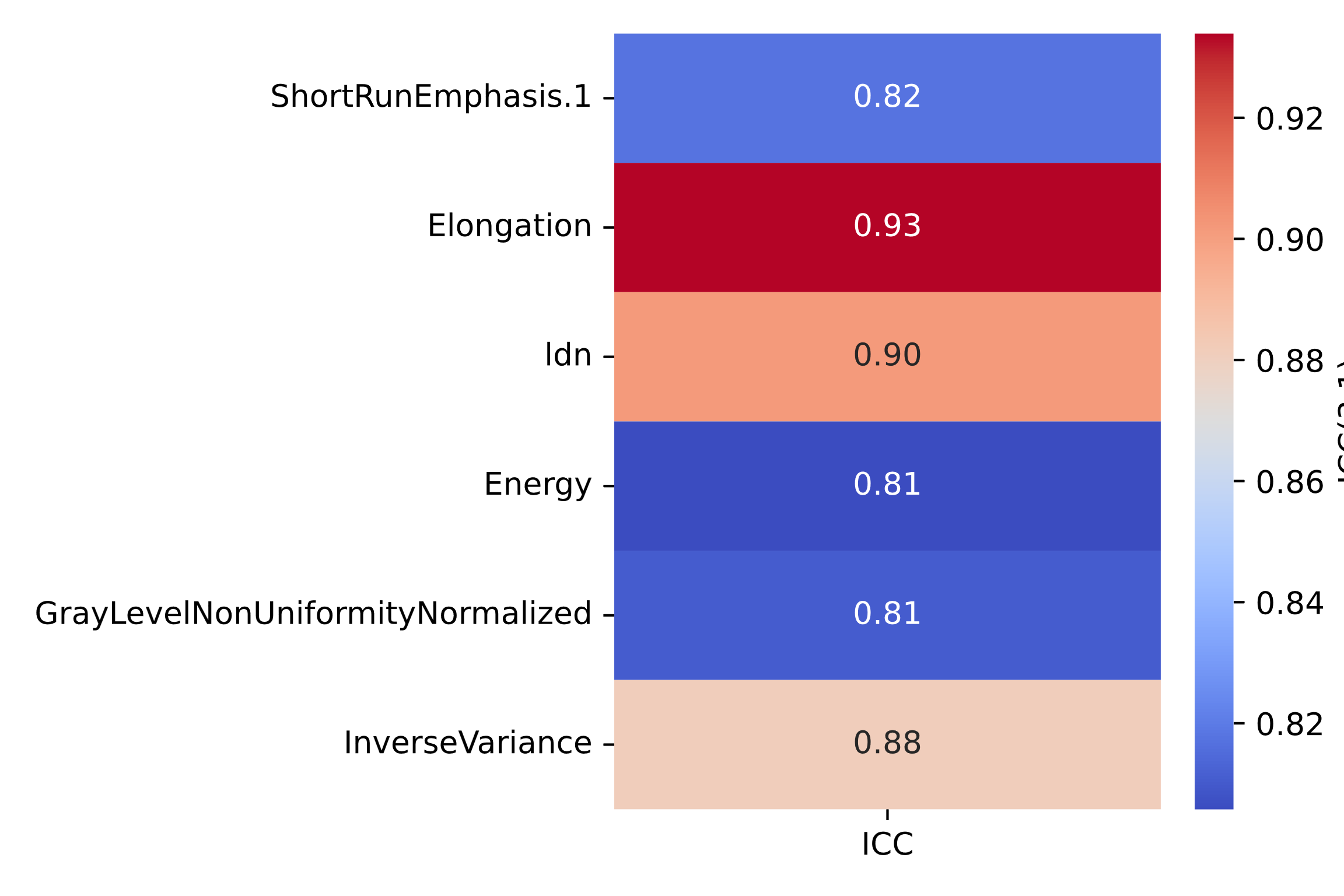

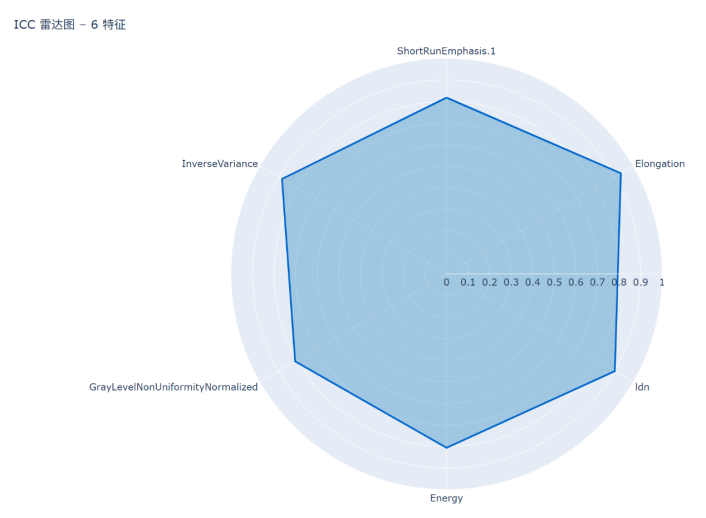


a

b

c

**Supplementary Figure 1** Visual ICC analysis of the final six selected features. **1a** The Y-axis shows six radiomics features — *Energy, GrayLevelNonUniformityNormalized, ShortRunEmphasis.1, InverseVariance, Idn,* and *Elongation* — representing classical texture- and shape-related descriptors. The X-axis denotes the intraclass correlation coefficient (ICC, range 0–1), with ICC > 0.75 indicating good agreement and ICC ≥ 0.80 considered a stricter benchmark.
**(1b, 1c)** Exact ICC values are displayed, enabling a detailed assessment of feature reproducibility. *Elongation* and *Idn* demonstrated excellent stability (ICC ≥ 0.90); *InverseVariance* and *ShortRunEmphasis.1* showed good agreement (ICC ≈ 0.85); while *Energy* and *GrayLevelNonUniformityNormalized* were threshold-compliant (ICC ≈ 0.81).
